# AHNAK deficiency promotes browning and lipolysis in mice via increased responsiveness to β-adrenergic signalling

**DOI:** 10.1038/srep23426

**Published:** 2016-03-18

**Authors:** Jae Hoon Shin, Seo Hyun Lee, Yo Na Kim, Il Yong Kim, Youn Ju Kim, Dong Soo Kyeong, Hee Jung Lim, Soo Young Cho, Junhee Choi, Young Jin Wi, Jae-Hoon Choi, Yeo Sung Yoon, Yun Soo Bae, Je Kyung Seong

**Affiliations:** 1 Laboratory of Developmental Biology and Genomics, Institute for Veterinary Science, and BK21 Program for Veterinary Science, College of Veterinary Medicine, Seoul National University, Seoul, Republic of Korea; 2 Korea Mouse Phenotyping Center (KMPC), Seoul National University, Seoul, Republic of Korea; 3 Department of Life Science, College of Natural Sciences, Research Institute for Natural Sciences, Hanyang University, Seoul, Republic of Korea; 4 Department of Anatomy and Cell Biology, College of Veterinary Medicine, Research Institute for Veterinary Science, Seoul National University, Seoul, South Korea; 5 Division of Life Sciences, Ewha Womans University, Seoul, Republic of Korea; 6 Interdisciplinary Program for Bioinformatics, Program for Cancer Biology, and Bio MAX Institute, Seoul National University, Seoul, Republic of Korea

## Abstract

In adipose tissue, agonists of the β3-adrenergic receptor (ADRB3) regulate lipolysis, lipid oxidation, and thermogenesis. The deficiency in the thermogenesis induced by neuroblast differentiation-associated protein AHNAK in white adipose tissue (WAT) of mice fed a high-fat diet suggests that AHNAK may stimulate energy expenditure via development of beige fat. Here, we report that AHNAK deficiency promoted browning and thermogenic gene expression in WAT but not in brown adipose tissue of mice stimulated with the ADRB3 agonist CL-316243. Consistent with the increased thermogenesis, *Ahnak*^−/−^ mice exhibited an increase in energy expenditure, accompanied by elevated mitochondrial biogenesis in WAT depots in response to CL-316243. Additionally, AHNAK-deficient WAT contained more eosinophils and higher levels of type 2 cytokines (IL-4/IL-13) to promote browning of WAT in response to CL-316243. This was associated with enhanced sympathetic tone in the WAT via upregulation of adrb3 and tyrosine hydroxylase (TH) in response to β-adrenergic activation. CL-316243 activated PKA signalling and enhanced lipolysis, as evidenced by increased phosphorylation of hormone-sensitive lipase and release of free glycerol in *Ahnak*^−/−^ mice compared to wild-type mice. Overall, these findings suggest an important role of AHNAK in the regulation of thermogenesis and lipolysis in WAT via β-adrenergic signalling.

Obesity is associated with several metabolic disorders, including type 2 diabetes, hypertension, cardiovascular disease and cancer^1^,^2^. Mammalian adipose tissue has been traditionally divided into two distinct types: white adipose tissue (WAT), which is the primary site of energy storage by accumulating lipids in unilocular (monovacuolar) adipocytes, and brown adipose tissue (BAT), which engages in energy expenditure through nonshivering thermogenesis and stores lipids in multilocular adipocytes^3^,^4^. Brown adipocytes generate heat through abundant mitochondria containing thermogenin or uncoupling protein 1 (UCP1) and are essential for the survival of small mammals in cold environments, and are considered an attractive target to counteract obesity. Recently, a distinct type of thermogenic fat cells termed “beige adipocytes” has been identified^5^,^6^. These beige fat cells exist within WAT but share common morphological and functional characteristics with classical brown adipocytes and can promote so-called “browning” of WAT and energy expenditure. Thus, there has been emerging interest in beige fat as a platform for anti-obesity therapeutic approaches^4^,^7^,^8^.

Several secreted factors, such as the peptide hormone irisin, bone morphogenetic protein 8B (BMP8b), thyroid prohormone thyroxine (T4), norepinephrine, and natriuretic peptides can induce browning in WAT^4^,^9^,^10^,^11^,^12^,^13^. Previous studies suggest that β-adrenergic signals produced by norepinephrine and β-adrenergic agonists can enhance lipolysis and adaptive thermogenesis^14^,^15^. Three adrenergic receptors mediate thermogenesis via sympathetic activation; among them, β3-adrenergic receptor (ADRB3) has been shown to alleviate obesity and diabetes and is abundant in rodent adipose tissue^16^,^17^. CL-316243, an ADRB3 agonist, has been utilized to mimic the browning effect in WAT of rodents^18^. In adipocytes, CL-316243 stimulates the production of cyclic AMP (cAMP), an activator of protein kinase A (PKA) that upregulates UCP1 and peroxisome proliferator-activated receptor gamma coactivator 1-alpha (PGC-1α), which is involved in thermogenesis^19^,^20^.

The neuroblast differentiation-associated protein AHNAK is a ubiquitously expressed high-molecular-weight protein essential for cell migration; it has been shown to regulate the actin cytoskeleton by functioning as a molecular linker for cardiac calcium channels^21^. We recently reported that *Ahank* knockout (KO) mice exhibit enhanced insulin sensitivity and higher energy expenditure under a high-fat diet^22^. The levels of UCP1 mRNA were increased in epididymal (e)WAT from high fat diet-fed *Ahnak*^−/−^ mice compared with that from wild-type mice. Although AHNAK is highly expressed in adipose tissue and up-regulated in murine models of obesity^23^,^24^, the thermogenic function of AHNAK in adipose tissue has not been determined.

In the present study, we demonstrate that loss of AHNAK leads to browning of WAT depot and promotes energy expenditure by β-adrenergic stimulation. *AHNAK* gene ablation upregulates mitochondria biogenesis and enhances lipolysis via induction of PKA-mediated gene expression in WAT in response to β-adrenergic signalling.

## Results

### Regulation of AHNAK in obesity

**Figure 1:**
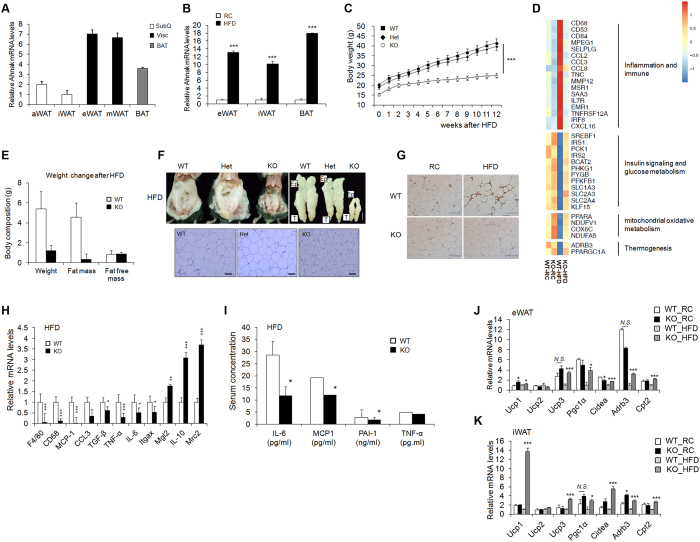
Effect of AHNAK ablation on fat tissue. (**A**) *AHNAK* mRNA expression determined by qPCR analysis of multiple adipose tissues from 10- to 12-week-old male wild-type mice (n = 4) and expression was normalized to that of 36B4. (**B**) *AHNAK* mRNA levels in eWAT, iWAT, and BAT from wild-type mice fed RC or HFD (n = 5–7 per group). (**C**) Body weight of male WT, heterozygotic (Het), and homozygotic knockout (KO) mice (n = 4–5 per group). (**D**) Representative gene expression profiles displaying differentially expressed genes in eWAT from RC-or HFD-fed WT and KO mice (full dataset available online as NCBI GEO data set GSE37218). (**E**) Changes in body composition of WT and KO mice fed HFD analysed by MRS (n = 7–9). (**F**) Representative images of epididymal fat depots and H&E staining of tissues from WT, hetero, and KO mice fed HFD. (**G**) Representative images of immunostaining of eWAT with an antibody against Mac-2. (**H**) mRNA expression of inflammation-related genes in mice fed HFD (n = 6–9). (**I**) Serum biochemistry of inflammation profiles in overnight-fasted mice fed HFD (n = 5–9). (**J,K**) mRNA expression of thermogenic genes in eWAT (**J**) and iWAT (**K**) from RC or HFD-fed WT and KO mice (n = 5). The data are presented as the mean ± SEM. *P < 0.05, **P < 0.01, ***P < 0.001, wild-type (WT) versus *Ahnak*^−/−^ (KO) mice.

AHNAK is involved in adipocyte differentiation and plays a crucial role in body fat accumulation^22^. To determine the expression of AHNAK in different fat depots, adipose tissues were classified as visceral (visc), subcutaneous (subQ), and brown fat (BAT). Notably, *AHNAK* expression was higher in visceral tissues compared to SubQ fat (Fig. 1A). *AHNAK* gene expression was significantly elevated in BAT and epididymal (eWAT) and inguinal (iWAT) fat depots in mice fed a high-fat diet (HFD) compared to mice fed a regular chow (RC) diet (Fig. 1B). In agreement with our previous data^22^, the *AHNAK* KO mice on the HFD exhibited decreased body weight when compared with wild-type and heterogygote knockout mice (Fig. 1C). Furthermore, *AHNAK* KO mice had significantly reduced body fat contents (Fig. 1E,F). Gene profiling of eWAT from HFD fed *AHNAK* KO mice showed increased insulin signalling and glucose metabolism. In contrast, inflammation-related genes were downregulated in HFD-fed *AHNAK* KO mice compared with their HFD-fed WT littermates (Fig. 1D). Consistently, HFD-induced macrophage infiltration in adipose tissue was less abundant in *AHNAK* KO mice (Fig. 1G). Expression of inflammatory genes, such as CD68, F4/80, monocyte chemoattractant protein-1 (MCP1), IL6, and TNF-α, was significantly decreased in eWAT of HFD-fed *AHNAK* KO mice with upregulation of M2 macrophage specific transcripts, including those encoding macrophage galactose N-acetyl-galactosamine specific lectin 2 (Mgl2), mannose receptor, C type 2 (Mrc2) and IL10 (Fig. 1H). Serum inflammatory parameters were also decreased in *AHNAK* KO mice (Fig. 1I).

We previously reported that AHNAK ablation increased energy expenditure in HFD-fed animals^22^. Consistently, thermogenic genes including Ucp1, Pgc-1α, Cidea, Cpt2, and Adrb3 were significantly upregulated in the eWAT and iWAT of HFD-fed *AHNAK* KO mice (Fig. 1J,K). However, there were no differences in BAT (Supplementary Fig. 1A). Notably, ablation of AHANK leads to upregulation of Adrb3, which is essential for the regulation of adaptive thermogenesis and oxidative metabolism^25^. These findings suggest that AHNAK ablation in WAT protects mice from obesity and its related complications accompanied by elevation of energy expenditure.

### AHNAK ablation promotes a thermogenic gene program and browning in WAT but not in BAT via β-adrenergic stimulation

To evaluate the role of *AHNAK* in cold-induced adaptive thermogenesis, mice were exposed to an ambient temperature of 4 °C for 3 days and compared to the animals kept at thermoneutral conditions (30 °C). *AHNAK* expression was selectively decreased in eWAT and iWAT (p < 0.07)(Supplementary Fig. 1B). At 30 °C, *AHNAK* KO mice had few UCP1-positive adipocytes and their morphology was similar to that of the wild-type mice. However, after cold exposure, *AHNAK* KO mice demonstrated an increase in multilocular UCP1-expressing adipocytes in eWAT and inguinal iWAT (Supplementary Fig. 2A). Both eWAT and iWAT but not BAT from AHNAK-deficient cold-exposed mice tended to have higher mRNA levels of thermogenic genes (*Pgc1*α, *Cidea, Dio2, Cpt1*); in addition, eWAT in these mice was enriched for beige adipocyte markers (*CD137, Tmem26*) compared to the wild-type mice after cold exposure (Supplementary Fig. 2B–D). These results suggest that *AHNAK* genetic ablation promotes adaptive browning of WAT under environmental stimulation.

**Figure 2:**
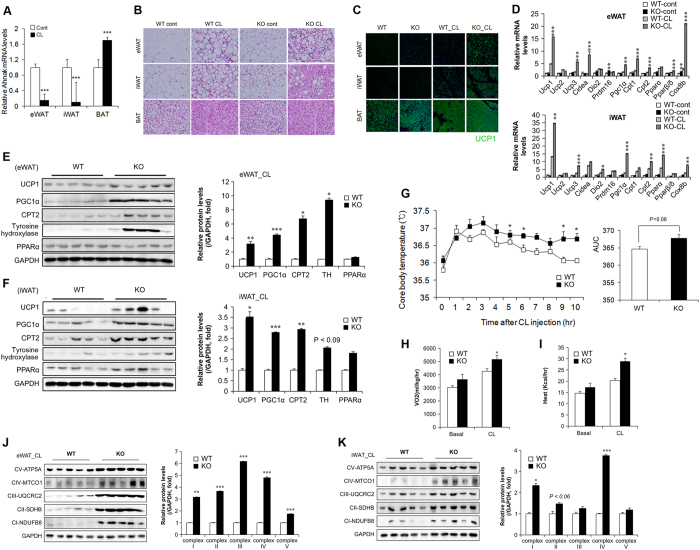
AHNAK ablation induces the transition of white to brown fat and enhances thermogenesis. (**A**) *AHNAK* mRNA expression of fat depots in control or CL-treated mice (n = 4–9 per group); expression was normalized to that of 36B4. (**B**) Representative images of H&E staining in various adipose tissues. (**C**) Anti-UCP1 (green) immunofluorescence staining of adipose tissues treated or not with CL CL-316243. (**D**) mRNA expression of thermogenic genes in eWAT and iWAT analysed by qPCR (n = 5). (**E,F**) Protein expression of thermogenic genes in response to CL-316243 injection in eWAT (E) and iWAT (**F**) analysed by immunoblotting; GAPDH was used as a loading control. (**G**) Core body temperature measured in WT and KO mice after CL injection at room temperature (n = 12). (**H,I**) VO_2_ (**H**) and heat generation (**I**) analysed by indirect calorimetry in WT and KO mice (n = 7 per group). (**J,K**) Immunoblotting analysis of mitochondrial respiratory chain complexes in eWAT (**J**) and iWAT (**K**) GAPDH was used as a loading control. The data are presented as the mean ± SEM. *P < 0.05, **P < 0.01, ***P < 0.001, wild-type (WT) versus *Ahnak*^−/−^ (KO) mice.

The sympathetic nervous system plays a critical role in BAT activation and adaptive thermogenesis mediated by β-adrenergic receptors^25^,^26^. To examine whether β-adrenergic signalling influenced thermogenesis, *AHNAK* KO mice were administered CL-316243 (CL), an ADRB3 agonist. When CL was administered to wild-type mice, we found significantly lower expression of *AHNAK* in the eWAT and iWAT, but not BAT (Fig. 2A). Histological analysis revealed numerous multilocular, UCP1-expressing adipocytes in the WAT of CL-treated *AHNAK* KO mice relative to wild-type controls. (Fig. 2B,C). We also examined cell proliferation in adipose tissue after CL treatment by BrdU labelling (Supplementary Fig. 3A). The frequency of BrdU^+^ cells expressing PDGFRα, a marker of adipocyte progenitors, was similar between WT and *AHNAK* KO mice under non-CL treatment (Supplementary Fig. 3B,C). We observed that 6.18 ± 0.77% of dividing cells expressed detectable adiponectin, a mature adipocyte marker that is constitutively expressed in non-CL-treated WAT of WT mice. This proportion was comparable to that of KO mice (6.64% ± 0.39) (Supplementary Fig. 3B.C). CL treatment increased the number of UCP1^+^ cells that incorporated BrdU (Supplement Fig. 3D,E). However, more UCP1^+^ cells were positive for BrdU in the CL-treated WAT of *AHNAK* KO mice than in that of WT mice (Supplementary Fig. 3D,E). These results indicate that the increase in UCP1^+^ adipocytes in the WAT of *AHNAK* KO mice is derived from increased proliferation of cells under β3 adrenergic stimulation. At the mRNA level, the expression of both brown adipocyte-related genes (Ucp1, Cidea, Dio2, and Pgc1α) and mitochondrial genes (Cpt1, Cpt2, and Cox8b) in CL-stimulated eWAT and iWAT was higher in *AHNAK* KO mice than in WT mice (Fig. 2D). Western blot analysis showed that UCP1, PGC1α, and CPT2 levels were significantly elevated in eWAT and iWAT from CL-stimulated *AHNAK* KO mice compared to that from WT mice (Fig. 2E,F). However, there were no obvious changes in the histological morphology and activity of BAT between WT and *AHNAK* KO mice (Fig. 2B, Supplementary Fig. 4A,B). Furthermore, *AHNAK* KO mice exhibited a higher core body temperature than wild-type mice after CL injection (Fig. 2G). Consistent with a previous report^27^, oxygen consumption (VO_2_) and energy expenditure were significantly elevated in *AHNAK* KO mice compared to WT mice after CL stimulation (Fig. 2H,I). These results suggest that β3-adrenergic stimulation mediates the formation of multilocular adipocytes and enhances adaptive thermogenesis through upregulation of thermogenic genes in the AHNAK-deficient WAT depots, leading to increased energy expenditure.

In addition, ADRB3 stimulation has been associated with reduction of fat mass and induction of mitochondrial biogenesis^28^. To determine whether the increase in energy expenditure and UCP1 expression in AHNAK-deficient WAT was associated with mitochondrial activation, we investigated the changes in mitochondrial biogenesis in adipose tissues. Expression of the OXPHOS genes in mitochondrial respiratory complexes I–V was remarkably increased in the WAT of CL-treated *AHNAK* KO mice compared to that of wild-type animals (Fig. 2J,K); however, no significant effect was observed for BAT (Supplementary Fig. 4C). Thus, the increased mitochondrial function indicated by the upregulation of OXPHOS genes may underlie the higher energy expenditure and metabolic activity in AHNAK-deficient WAT.

### Increased levels of eosinophils and type 2 cytokines in AHNAK-deficient WAT

**Figure 3:**
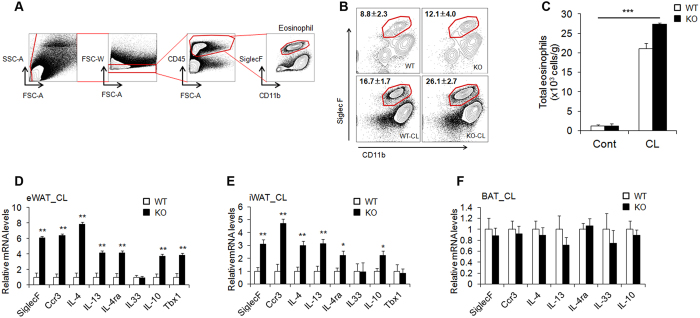
Increased levels of eosinophils and type 2 cytokines in *AHANK*-deficient WAT. (**A**) Gating strategy for identification of eosinophil lineage defined as CD45^+^ CD11b^+^ Siglec F^+^. (**B,C**) Representative plots and frequencies (**B**) and number (**C**) of eosinophils in eWAT of mice treated with CL-316243 (n = 4 group pooled from 7–10 mice). Statistical analysis was performed using the Kruskal-Wallis test (***P < 0.001) (**D–F**) qPCR analysis of eosinophil- and type 2 cytokine-related genes in eWAT (**D**), iWAT (**E**), and BAT (**F**) of mice treated with CL-316243 (n = 5–7); expression was normalized to that of 36B4. Data are the mean ± SEM; *P < 0.05, **P < 0.01, ***P < 0.001.

We observed phenotypic change into brown-like adipocytes in CL-stimulated WAT, even though we used mice with a null mutation. We therefore hypothesize that this enhanced thermogenesis through β-adrenergic signalling can be induced by non-adipose progenitor cell types. Recent studies have focused on the eosinophil-mediated regulation of beige fat development^29^,^30^,^31^. To investigate whether or not eosinophils mediate WAT browning induced by β-adrenergic stimulation, we isolated eWAT and identified cell lineages that express sialic acid-binding immunoglobulin receptor (Siglec-F) and CD11b as molecular markers for eosinophils (Fig. 3A). CL exposure increased the frequency and number of Siglec-F^+^/CD11b^+^ cells, representing eosinophils, in AHNAK-deficient eWAT (Fig. 3B,C). Consistently, mRNA levels of eosinophil (Siglec-F and Ccr3), type 2 cytokines (IL-4, IL-4ra, and IL-13), and an alternatively activated M2-specific gene (IL-10) were also significantly increased in the eWAT and iWAT of CL-treated *AHNAK* KO mice (Fig. 3D,E), but not in the BAT (Fig. 3F). Eosinophils and type 2 cytokines are implicated in regulation of the biogenesis of thermogenic beige fat^30^,^32^. Together, these results indicate that genetic ablation of *AHNAK* augments eosinophil content and type 2 cytokine signals in CL-treated WAT, which could promote WAT browning upon β3-adrenergic stimulation.

### Ablation of AHNAK increases fat lipolysis

**Figure 4:**
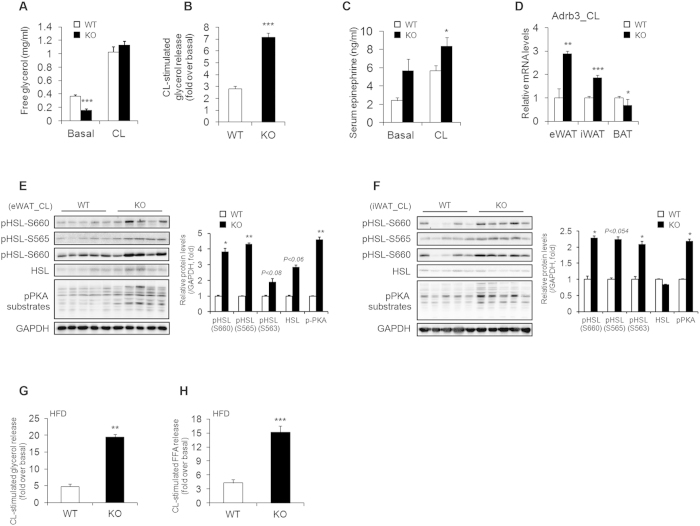
Enhanced β-adrenergic signalling in WAT of *Ahnak*^−^/^−^ mice. (**A**) CL-316243 stimulated glycerol levels in serum from RC-fed mice (20 min after CL injection) (n = 5). (**B**) Fold changes of glycerol levels over basal level after CL316243 stimulation in serum (n = 5). (**C**) Serum epinephrine levels (n = 7–9 for basal, n = 17–20 for CL). (**D**) Relative mRNA levels of Adrb3, assessed by qRT-PCR in CL-treated adipose tissue (n = 5); the expression was normalized to that of 36B4. (**E,F**) Levels of phosphorylated HSL and PKA substrates assessed by immunoblotting in eWAT (**E**) and iWAT (**F**) GAPDH was used as a loading control. (**G,H**) CL-316243 stimulated glycerol (**G**) and FFA (**H**) levels in serum from HFD-fed mice (20 min after CL injection) (n = 4–5). The data are presented as the mean ± SEM; *P < 0.05, **P < 0.01, ***P < 0.001, wild-type (WT) versus *Ahnak*^−/−^ (KO) mice.

Stimulation by a β3-adrenergic receptor agonist induces activation of protein kinase A (PKA), which then leads to lipolysis mediated by phosphorylation of hormone-sensitive lipase (HSL)^33^. Therefore, we investigated catecholamine signalling in the adipose tissue of CL-treated *AHNAK* KO mice. There was no difference in the serum concentration of free glycerol between *AHANK* KO and wild-type mice after CL injection, probably because of a decrease in body fat in *AHNAK* KO mice (Fig. 4A). However, exposure to CL316243 resulted in a higher fold increase in glycerol release in *AHNAK* KO mice than in wild-type mice (Fig. 4B). Importantly, AHNAK-deficient WAT also displayed increased expression of tyrosine hydroxylase, the rate-limiting enzyme in the synthesis of catecholamines (Fig. 2E,F). Moreover, circulating levels of epinephrine were also increased in KO mice (Fig. 4C), suggesting that white fat browning may be attributed to enhanced sympathetic input to WAT in AHNAK-deficient mice. Furthermore, Adrb3 mRNA expression was significantly increased after CL stimulation in WAT but not in BAT of *AHNAK* KO mice (Fig. 4D). However, the expression of other adrenergic receptor subtypes, i.e., Adrb1 and Adrb2, was not altered (Supplementary Fig. 5A,B). As previously decribed^34^, the levels of Rgs2, a negative regulator of adrenergic signalling, were also not different between the two groups (Supplementary Fig. 5C). These results suggest that robust sympathetic tone and enhanced catecholamine sensitivity may alter β-adrenergic responsiveness in AHNAK-deficient WAT. In support of this notion, PKA activity in WAT was substantially elevated in *AHNAK* KO mice as evidenced by the increase in the level of phosphorylated PKA substrates and hormone sensitive lipase (HSL) in both eWAT and iWAT of AHNAK-deficient mice after CL injection (Fig. 4E,F); however, no changes were observed in BAT (Supplementary Fig. 5D). Furthermore, *AHNAK* KO mice exhibited a higher CL-stimulated lipolytic response after HFD feeding (Fig. 4G,H). Thus, AHNAK may regulate catecholamine sensitivity in WAT.

**Figure 5:**
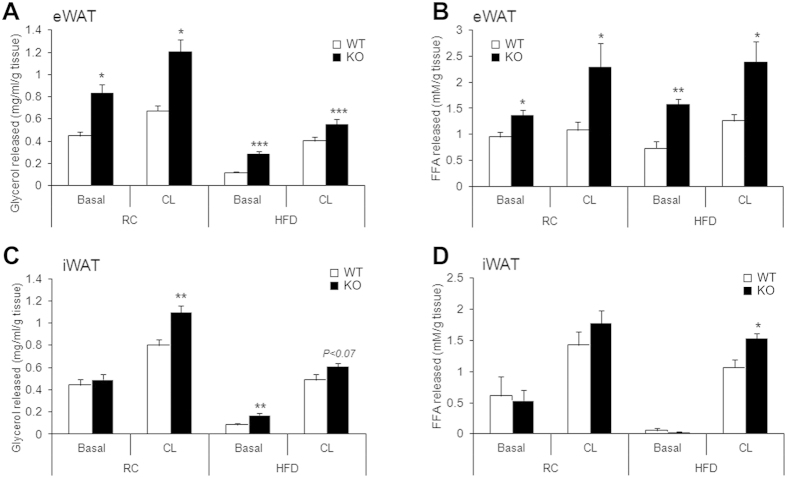
AHNAK deficiency enhances lipolysis in adipocytes *ex vivo* in response to β-adrenergic stimulation. (**A**) Released glycerol and (**B**) FFA from explants of RC or HFD-fed eWAT (**C**) Glycerol and (**D**) FFA released from explants of RC or HFD-fed iWAT (n = 4–6 per group). The values were normalized to the wet weight of the explants. The data are presented as the mean ± SEM; *P < 0.05, **P < 0.01, ***P < 0.001, wild-type (WT) versus *Ahnak*^−/−^ (KO) mice.

Mice lacking AHNAK exhibited enhanced β-adrenergic signalling in WAT; therefore, we examined the effect of AHNAK on acute adrenergic signalling in murine adipose tissue and adipocytes. Isolated adipose tissues were incubated with CL for 2 h at 37 °C, and the release of FFA and glycerol to media was determined and normalized to the fat tissue weight. In both eWAT and iWAT of AHNAK-deficient mice under either RC or HFD feeding, CL treatment led to significantly increased lipolysis, as indicated by FFA and free glycerol release (Fig. 5A–D). Taken together, these results suggest that fat increased lipolysis in AHNAK ablation is mediated by β3-adrenergic signalling through induction of Adrb3.

## Discussion

Understanding of signalling pathways that regulate thermogenesis and enhance energy expenditure and identification of their potential targets is important for the development of measures to control obesity. It has been suggested that activation of brown adipocytes and increased BAT mass may constitute effective strategies to counteract obesity^7^,^8^. Recent studies have reported that catecholamine resistance in adipose tissue could be associated with obesity^35^,^36^. AHNAK-deficient mice were previously shown to display increased energy expenditure during HFD challenge, indicating resistance to obesity^22^. Here, we revealed a critical role of AHNAK in regulating browning of WAT in response to β-adrenergic stimulation. AHNAK deficiency promotes an increase in the number of functional and proliferating brown-like adipocytes in WAT depots, which results in higher energy expenditure following β3-adrenergic stimulation. Regardless, the proliferation and differentiation of progenitors in AHNAK deficiency still remains to be determined and traced *in vivo*.

ADRB3 agonists have been recognized as critical mediators of lipolysis and thermogenesis. ADRB3 activation stimulates cAMP and PKA activation, which promotes adipocyte lipolysis via HSL phosphorylation^33^. ADRB3-activated PKA stimulates the transcriptional coactivator PGC1α, leading to increased UCP1 expression and thermogenesis^19^,^37^. Efforts have been made to develop therapeutic approaches targeting ADRB3 to treat or prevent obesity and metabolic disorders, and CL-316243 has been used for generating brown-like adipocytes in WAT^17^,^38^. However, the development of such drugs has been challenging because of insufficient oral bioavailability and cross-reactivity with ADRB1^39^,^40^. Nevertheless, new ADRB3 agonists have been investigated in clinical trials and have shown improved bioavailability and binding affinity^41^,^42^,^43^. In this study, AHNAK-deficient CL-316243-stimulated mice demonstrated PKA activation in WAT, which was accompanied by increased phosphorylation of HSL and FFA mobilization, indicating enhanced lipolysis. Moreover, genetic ablation of *AHNAK* increased the expression of genes involved in fat browning (*Ucp1, Pgc1a*, and *Cpt2*) and mitochondrial oxidative phosphorylation in response to β-adrenergic signalling. Our results suggest that AHNAK deficiency may contribute to improved catecholamine sensitivity by enhancing ADRB3 expression and signalling in WAT.

Several factors have been reported to be involved in fat browning and thermogenesis. PGC1α has been recognized as a key regulator of energy metabolism and mitochondrial biogenesis. PGC1α activation regulated by mitogen-activated protein kinase p38 enhances the expression of thermogenic genes in adipose tissues in response to β-adrenergic signaling^44^. It has been shown that *Pgc1*α^−/−^ mice exhibit an abnormal thermogenic phenotype, suggesting that PKA activation, which regulates PGC1α expression, mediates thermogenesis^45^. CPT2 is a crucial regulator of long chain fatty acid transport and is involved in initiation of β-oxidation in mitochondria. Mice overexpressing the vitamin D receptor were previously shown to have reduced energy metabolism, which correlated with decreased fatty acid oxidation and lipolysis, in addition to suppression of CPT2 in WAT^46^. Mice with adipose tissue-specific CPT2 ablation are defective in cold- or CL-induced thermogenesis because of deregulation of fatty acid oxidation, suggesting that the increase in CPT2 underlies the enhanced lipolysis and thermogenesis in *AHNAK* KO mice^47^.

A local thermogenic circuit involving eosinophils and type 2 cytokines activates WAT browning and induces thermogenesis^29^,^30^, whereas inhibition of tyrosine hydroxylase, a rate-limiting enzyme of catecholamine biosynthesis, or IL-4/13 signalling disrupts browning-related responses^30^,^48^. Here, we investigated the process of generating functional beige cells in AHNAK-deficient WAT. AHNAK ablation led to robust recruitment of eosinophils into WAT and induced the secretion of eosinophil-derived cytokines after β-adrenergic stimulation, resulting in the production of epinephrine accompanied by increased expression of IL-10 and tyrosine hydroxylase, which promotes WAT browning and enhances mitochondrial biogenesis. It is still unclear whether eosinophils are regulated in an AHNAK-dependent manner because these parameters are also increased in unstimulated AHNAK-deficient eWAT. These results suggest that the increase in eosinophils and cytokine (IL-4/IL-13) secretion by adipocyte precursors could lead to catecholamine-induced WAT browning in *AHNAK* KO mice.

Collectively, our results demonstrate that the enhanced response to β-adrenergic signalling in *AHNAK* KO mice induces the development of beige fat, which promotes thermogenic effects with minimal alteration in BAT activity and mass. Although the precise molecular mechanism underlying AHNAK involvement in β-adrenergic signalling remains to be determined, downregulation of AHNAK may be a new approach to increase the response to β-adrenergic stimulation and energy expenditure in adipose tissue, thereby treating obesity.

## Methods

### Animals

*AHNAK* KO mice were generated by disruption of exon 5 in the *Ahnak* gene, as previously described^49^. Mice were maintained under a 12-h light/dark cycle at 22–24 °C in a specific pathogen-free barrier facility and had free access to water and a regular chow diet or a high-fat diet (HFD; 20% carbohydrate, 60% fat, 20% protein; D12492; Research Diets Inc., New Brunswick, NJ, USA). During the cold challenge experiments, mice (3 per cage) fed ad libitum were maintained in a 4 °C chamber (Daehan Biolink, Chungbuk, South Korea) for 3 days. To stimulate browning of white adipose depots, 2–3 month-old mice received daily intraperitoneal (i.p.) injections of 1 mg/kg body weight CL-316243 (Sigma-Aldrich, St. Louis, MO, USA) in PBS. BrdU (Sigma) was injected i.p. daily at a concentration of 50 mg/kg. For EdU flash-labelling, we used the Click-iT EdU Alexa Fluor 594 imaging kit (Invitrogen, Carlsbad, CA, USA). Mice were injected with EdU (4 nmol/mouse i.p.) 2 h before analysis and EdU was detected according to the manufacturer’s instructions. The experiments were performed according to the “Guide for Animal Experiments” (Edited by Korean Academy of Medical Sciences) and approved by the Institutional Animal Care and Use Committee (IACUC) of the Seoul National University (Permit Number: SNU-130903-1).

### Indirect calorimetry

Oxygen consumption (VO_2_), carbon dioxide production (VCO_2_), respiratory exchange ratios (RER), and heat production were measured using an indirect calorimetry system (TSE Systems, Bad Homburg, Germany). Mice in each chamber were maintained at a constant environmental temperature of 22 °C.

### Core body temperature measurement

Body temperature was measured using telemetry transmitters (TSE Systems). A transmitter was implanted into the peritoneal cavity under anaesthesia.

### Immunostaining

Adipose tissue samples were fixed in 4% paraformaldehyde, embedded in paraffin, sectioned, and preincubated with quenching buffer (3% H_2_O_2_ in methyl alcohol) and blocking buffer (10% normal goat serum in PBS) for 30 min at room temperature. The samples were then treated with the primary antibodies against adiponectin (1:100, Thermo Scientific, IL, USA), PDGFRα (1:50, R&D System, MN, USA), UCP1 (1:300, Abcam, Cambridge, UK) and BrdU (1:100, Abcam) and with secondary Alexa Fluor 488- or 594-conjugated antibodies (Invitrogen). To detect BrdU staining, paraffin sections were incubated in 50% formamide 2× SSC (Welgene Inc., Daegu, Korea) for 2 h at 65 °C, washed with 2× SSC, and treated with 2 N HCl for 30 min at 37 °C. After washing with PBS, tissue sections were blocked with 10% normal goat serum (Vector Laboratories CA, USA). For nuclear staining, the samples were mounted on cover slips in DAPI-containing mounting medium (ImmunoBioScience, Burlingame, WA, USA). Images were captured using a confocal microscope system (LSM 710, Carl Zeiss, Oberkochen, Germany).

### Western blotting

Total proteins were extracted using PRO-PREP buffer (iNtRON Biotechnology Inc., Seoul, Korea) containing phosphatase inhibitors (GenDEPOT, Barker, TX, USA), separated by SDS-PAGE, transferred to PVDF membranes (Millipore, Billerica, MA, USA), and analysed by immunoblotting. Primary antibodies against the following proteins were used: UCP1, PGC1α, CPT2, PPARα, AHNAK, and OXPHOS (oxidative phosphorylation) complexes (all from Abcam); pHSL, HSL, pPKA substrates, and GAPDH (all from Cell Signaling Technology, Beverly, MA, USA); and tyrosine hydroxylase (Millipore). The antibodies were diluted 1:1,000 with TBS containing 0.1% (v/v) Tween-20 (TBST, Biosesang, Seongnam, Korea). The membranes were then incubated with a peroxidase-conjugated secondary antibody (AbClon, Seoul, Korea), and antibody-specific signals were detected by enhanced chemiluminescence and quantified using the MicroChemi 4.2 system (DNR Bio Imaging Systems, Jerusalem, Israel).

### Quantitative real-time PCR

Total RNA was extracted using an RNA purification system (Invitrogen) following the manufacturer’s protocol. mRNA was reverse-transcribed using AccuPower CycleScript RT PreMix (Bioneer, Daejeon, Korea) and quantitative (q)PCR was performed using SYBR Green and an ABI StepOne Real-Time PCR instrument (Applied Biosystems, Cheshire, UK). Target gene expression was normalized to that of the control gene (36B4), and relative expression was quantified by the comparative Ct method (ΔΔCt). The primers used for PCR are listed in Supplementary Table 1.

### Lipolysis assay

To assess *in vivo* lipolysis, serum free fatty acids (FFAs) were measured in cardiac blood obtained from non-fasted mice before and 20 min after i.p. injection of CL316243 (1 mg/kg). *Ex vivo* and *in vitro* lipolysis was measured as described previously^36^,^50^ with some modifications. Briefly, for *ex vivo* analysis, adipose tissues were surgically removed from male and female mice, weighed, and washed with cold PBS; fat depots were incubated with CL316243 (1 μM) or vehicle in Krebs-Ringer Bicarbonate Buffer (KRBH, Sigma) containing 1% fatty acid-free BSA (GenDEPOT) and glucose (2.5 mM) for 2 h at 37 °C with mild shaking. After incubation, FFA and glycerol were measured using a glycerol-free reagent (Sigma) and the FFA quantification kit (BioVision, Mountain View, CA, USA) and normalized to the weight of adipose tissue samples.

### Biochemical assay

Serum epinephrine levels were determined using a commercially available ELISA kit according to the manufacturer’s instructions (Abnova, Taipei, Taiwan). Serum triglycerides, total cholesterol, HDL-cholesterol and LDL-cholesterol concentrations were analysed by Cobas c111 (Roche). Adipokines including adiponectin, leptin, IL-6, MCP1, PAI-1, and TNF-α were measured using MILLIPLEX MAP kits (Merck Millipore, Germany) according to the manufacturer’s instructions.

### Cell preparations and flow cytometric analysis

Epididymal white adipose tissues were chopped with scissors for 4 minutes, followed by addition of 2 mL of collagenase II (SIGMA C6885, 400 U/mL) and incubation at 37 °C for 8 minutes. After digestion, single-cell suspensions were treated with 20 μL of 0.5 M EDTA and filtered using 70 μm meshes. The cells were centrifuged at 385 *g* for 10 minutes at 4 °C to remove floating adipocytes, and red blood cells were lysed in 1× RBC lysis buffer (eBioscience 00-4300-54) for 2 minutes on ice. After blocking of Fc receptors using TruStain FcX (Biolegend 101320), the cells were stained with the indicated fluorochrome conjugated antibodies. We used a LSRFortessa (BD) or FACSCanto II (BD) system, and analysed flow cytometric data using FlowJo (Tree Star Inc.). The antibodies used for flow cytometry were PE-conjugated anti-CD64 (FcγRI) (Biolegend, 139304), PE-conjugated anti-IgG2a,κ (Biolegend, 400507), PerCP-conjugated anti-CD45 (Biolegend, 103130), PE/cy7-conjugated anti-CD11c (Biolegend, 117318), APC-conjugated anti-CD11b (Biolegend, CD11b), and BV421-conjugated anti-SiglecF (BD Horizon, 562681).

### Statistics

All values were expressed as the mean ± SEM. Statistical analysis was performed using Student’s t-test between two groups. The Kruskal-Wallis test was used for flow cytometric analysis. P < 0.05 was considered significant.

## Additional Information

**How to cite this article**: Shin, J. H. *et al*. AHNAK deficiency promotes browning and lipolysis in mice via increased responsiveness to β-adrenergic signalling. *Sci. Rep.* **6**, 23426; doi: 10.1038/srep23426 (2016).

## Supplementary Material

Supplementary Information
