# Supplementary material for: AHNAK deficiency promotes browning and lipolysis in mice via increased responsiveness to β-adrenergic signalling
Source: Sci Rep. 2016 Mar 18;6:23426. doi: 10.1038/srep23426 (PMC4796812; doi:10.1038/srep23426)
Supplement: Supplementary Information [file srep23426-s1.doc]

**AHNAK deficiency promotes browning and lipolysis in mice via increased responsiveness to β-adrenergic signalling**

Jae Hoon Shin^1,2^, Seo Hyun Lee^1,2^, Yo Na Kim^1,2^, Il Yong Kim^2^, Youn Ju Kim^1,2^, Dong Soo Kyeong^1,2^, Hee Jung Lim^2^, Soo Young Cho^2^, JunHee Choi^3^, Young Jin Wi^3^, Jae-Hoon Choi^3^, Yeo Sung Yoon^4^, Yun Soo Bae^5^ & Je Kyung Seong^1,2,6*^

^1^Laboratory of Developmental Biology and Genomics, Institute for Veterinary Science, and BK21 Program for Veterinary Science, College of Veterinary Medicine, Seoul National University, Seoul, Republic of Korea

^2^Korea Mouse Phenotyping Center (KMPC), Seoul National University, Seoul, Republic of Korea

^3^Department of Life Science, College of Natural Sciences, Research Institute for Natural Sciences, Hanyang University, Seoul, Republic of Korea

^4^Department of Anatomy and Cell Biology, College of Veterinary Medicine, Research Institute for Veterinary Science, Seoul National University, Seoul, South Korea

^5^Division of Life Sciences, Ewha Womans University, Seoul, Republic of Korea

^6^Interdisciplinary Program for Bioinformatics, Program for Cancer Biology, and Bio MAX Institute, Seoul National University, Seoul, Republic of Korea

Correspondence to: Je Kyung Seong ([snumouse@snu.ac.kr](mailto:snumouse@snu.ac.kr))

**Figure S1**


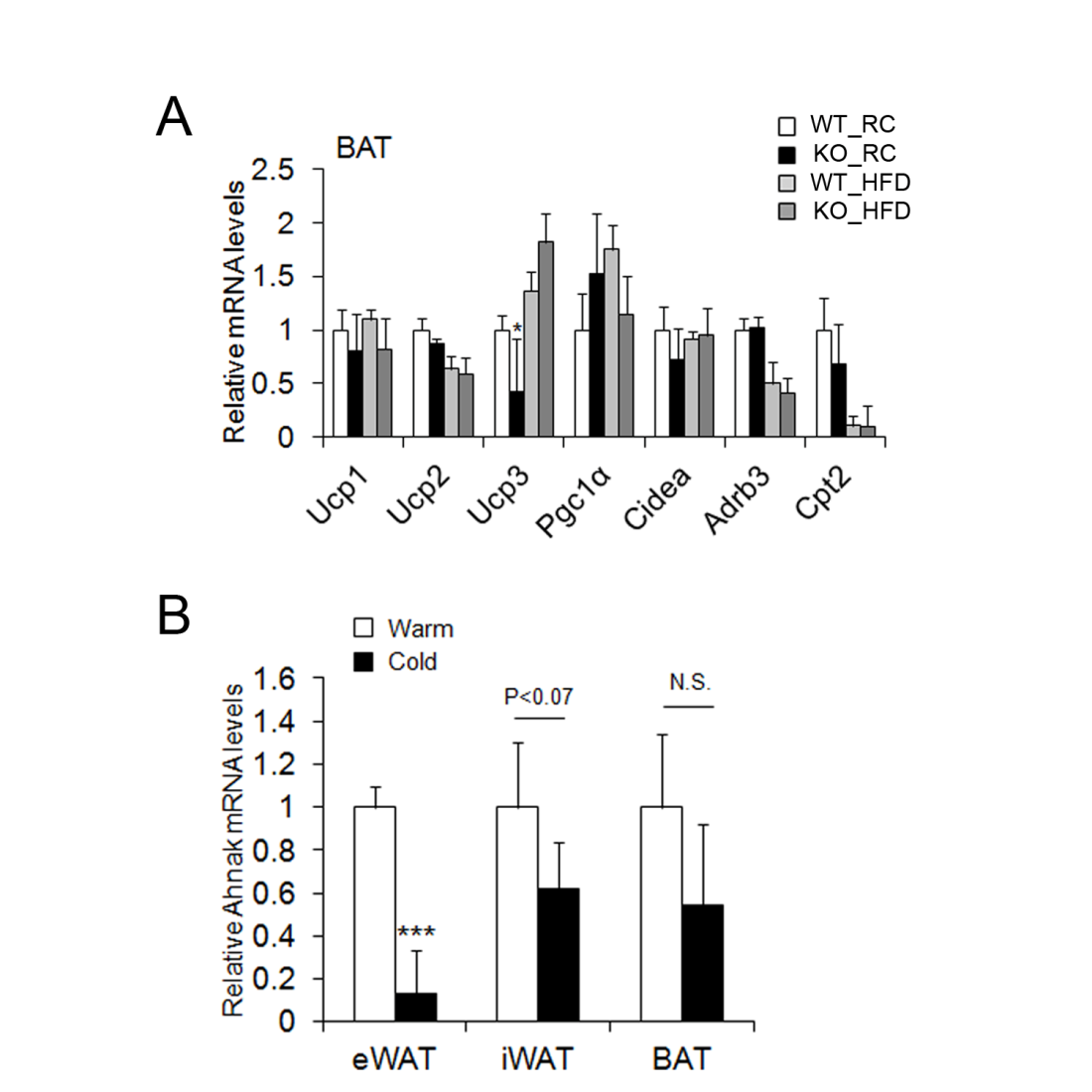


**Figure S2**


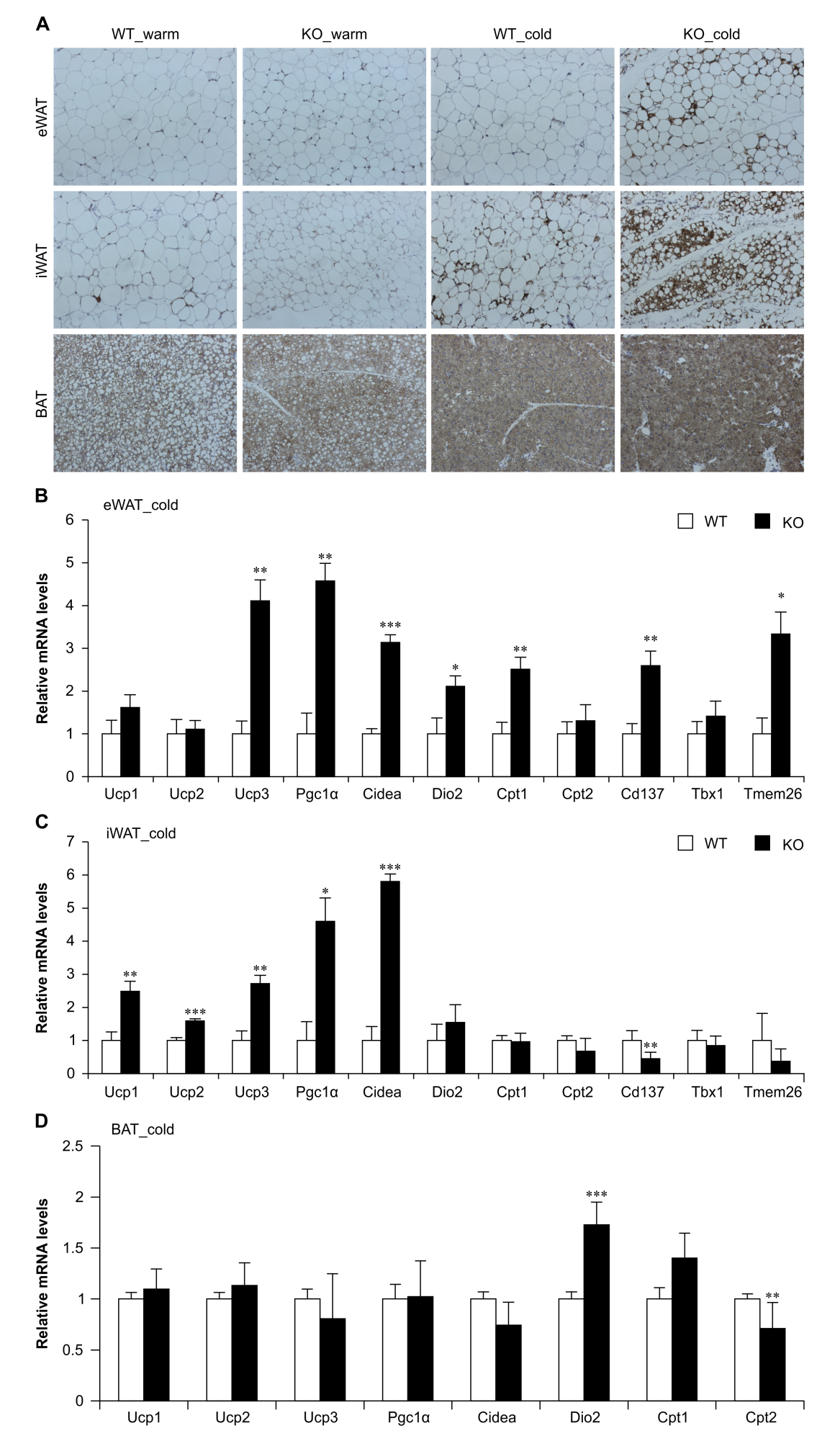


**Figure S3**

**
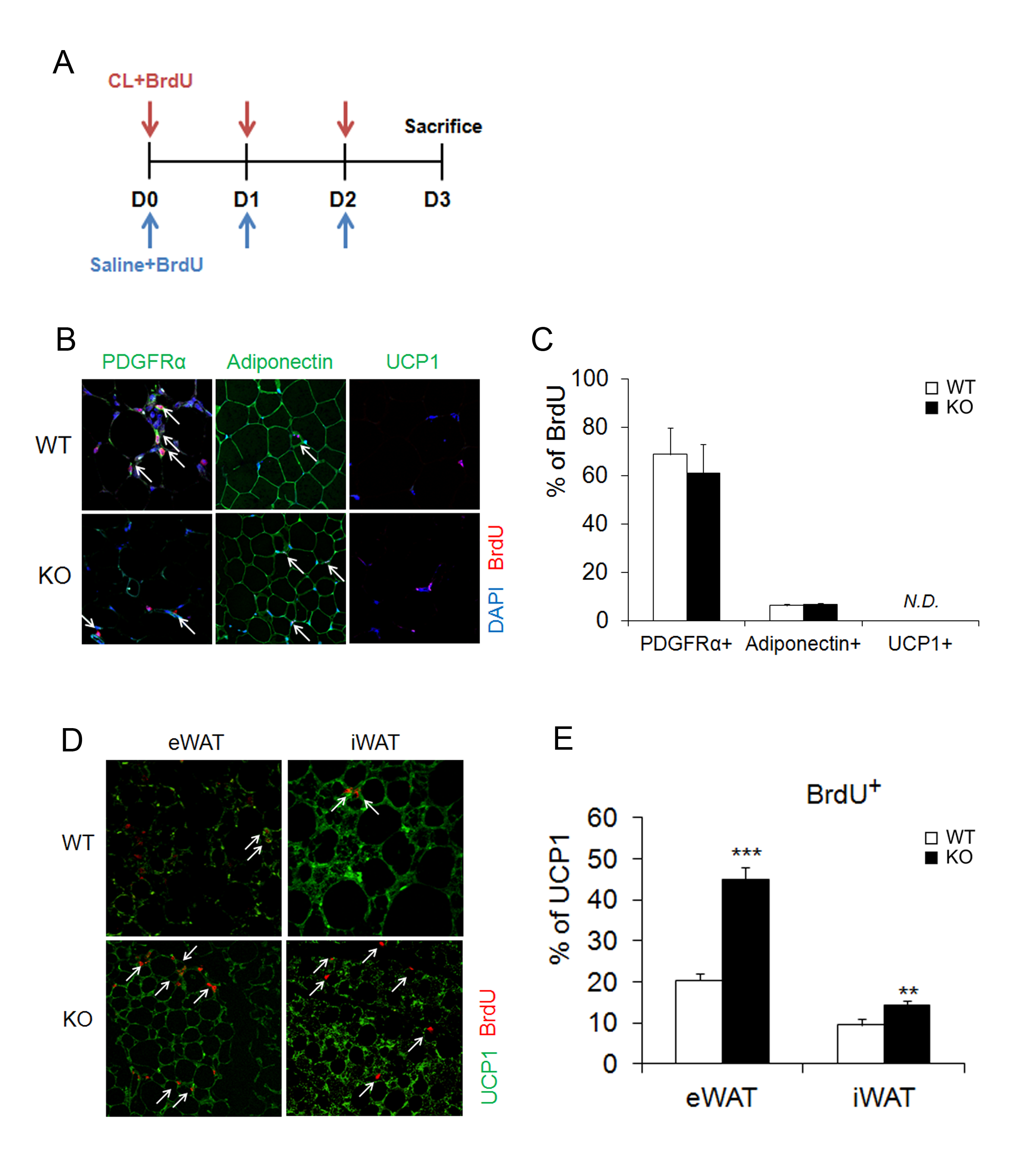
**

**Figure S4**


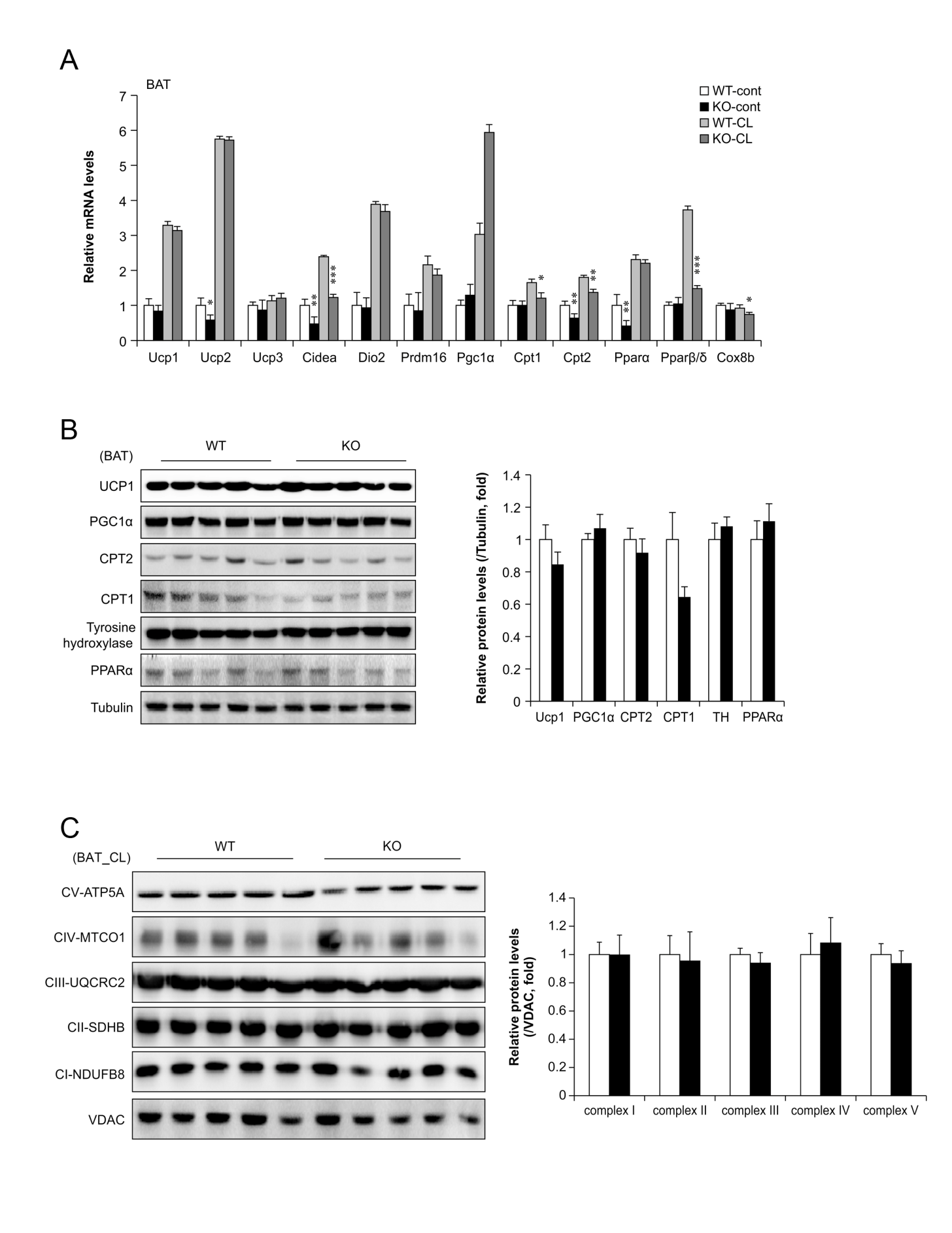


**Figure S5**

**
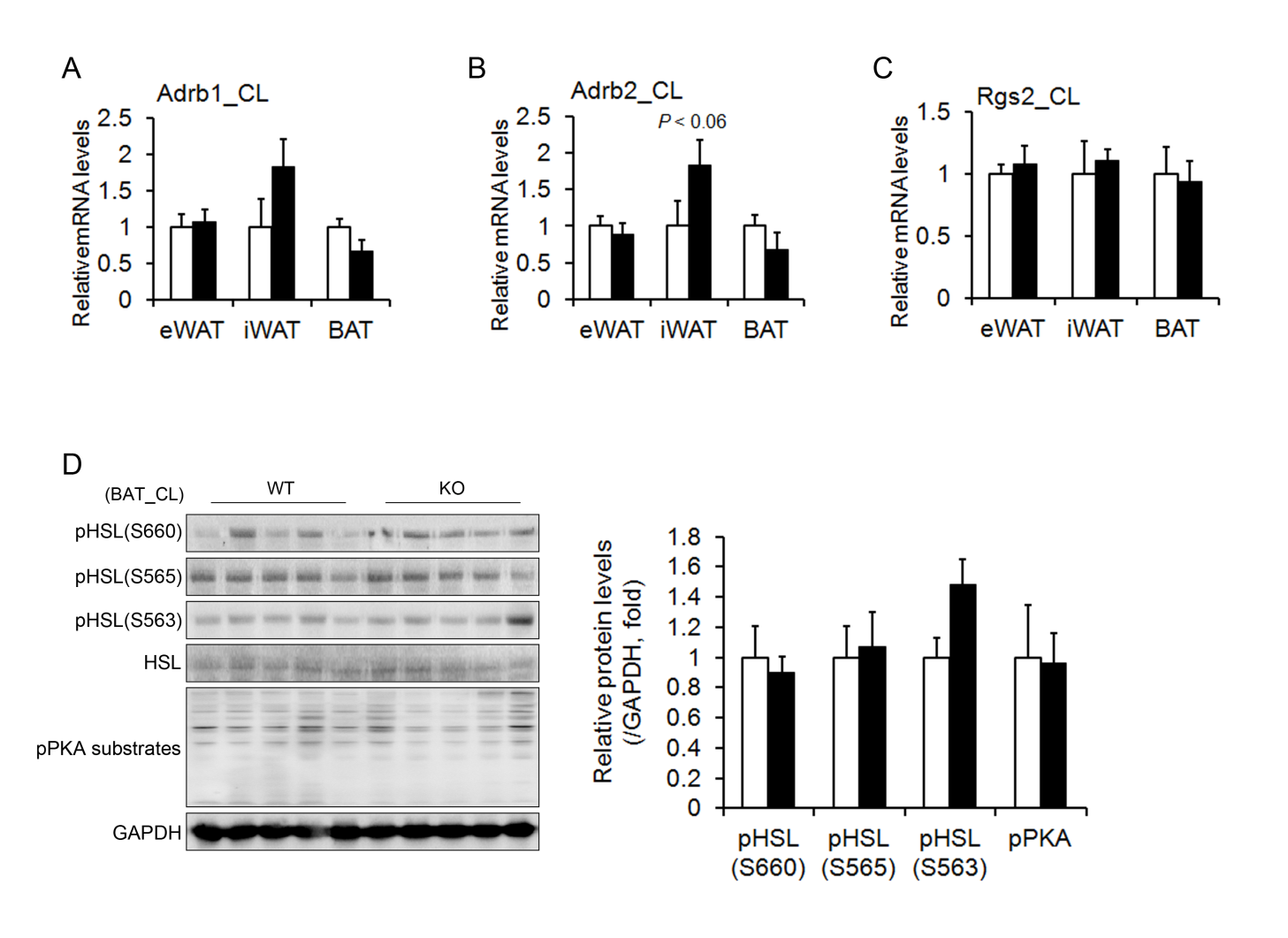
**

**Supplementary Table 1.** Primer sequences for quantitative PCR

| **Gene** | **Sequence** | **Gene** | **Sequence** |
| --- | --- | --- | --- |
| *Adrb1* | CTCATCGTGGTGGGTAACGTG | *IL-13* | CCTGGCTCTTGCTTGCCTT |
|  | ACACACAGCACATCTACCGAA |  | GGTCTTGTGTGATGTTGCTCA |
| *Adrb2* | GGGAACGACAGCGACTTCTT | *Pparα* | TCGGCGAACTATTCGGCTG |
|  | GCCAGGACGATAACCGACAT |  | GCACTTGTGAAAACGGCAGT |
| *Adrb3* | ATTGGCGCTGACTGGCCATT | *Pparβ/δ* | TTGAGCCCAAGTTCGAGTTTG |
|  | CCTCGGCATCTGCCCCTACA |  | CGGTCTCCACACAGAATGATG |
| *Ccr3* | TCGAGCCCGAACTGTGACT | *Prdm16* | CAGCACGGTGAAGCCATTC |
|  | CCTCTGGATAGCGAGGACTG |  | GCGTGCATCCGCTTGTG |
| *Cidea* | TGCTCTTCTGTATCGCCCAGT | *Pgc1α* | CCCTGCCATTGTTAAGACC |
|  | GCCGTGTTAAGGAATCTGCTG |  | TGCTGCTGTTCCTGTTTTC |
| *Cox8b* | GAACCATGAAGCCAACGACT | *Rgs2* | GAGAAAATGAAGCGGACACTCT |
|  | GCGAAGTTCACAGTGGTTCC |  | GCAGCCAGCCCATATTTACTG |
| *Cpt1* | TCTATGAGGGCTCGCG | *SiglecF* | CTGGCTACGGACGGTTATTCG |
|  | CGTCAGGGTTGTAGCA |  | GGAATTGGGGTACTGGACTTG |
| *Cpt2* | GCCCAGCTTCCATCTTTACT | *Ucp1* | ACTGCCACACCTCCAGTCATT |
|  | CAGGATGTTGTGGTTTATCCGC |  | CTTTGGCTCACTCAGGATTGG |
| *Dio2* | CAGTGTGGTGCACGTCTCCAATC | *Ucp2* | CAGATGTGGTAAAGGTCCGCT |
|  | TGAACCAAAGTTGACCACCAG |  | TTCCTCTCGTGCAATGGTCTT |
| *IL-4* | GGTCTCAACCCCCAGCTAGT | *Ucp3* | GAGATGGTGACCTACGACATCA |
|  | GCCGATGATCTCTCTCAAGTGAT |  | GCGTTCATGTATCGGGTGTTTA |
| *IL-4Rα* | GTCACAGAGCAGCCTTCACA | *36B4* | GAGGAATCAGATGAGGATATGGGA |
|  | AAAACTCCGGTAGGCAGGAT |  | AAGCAGGCTGACTTGGTTGC |

**Supplementary Figure legends**

Figure S1. (A) qPCR analysis of thermogenesis-related genes in BAT from RC and HFD-fed mice (n=5). (B) Expression levels of Ahnak gene in fat depots (n=5). Values were normalized to those of 36B4. Data are the mean ± SEM; *P<0.05, **P<0.01, ***P<0.001.

Figure S2. (A) Representative images of UCP1 immunostaining in adipose tissues from mice maintained at 30°C (warm) or 4°C (cold) for 3 days, and from mice with cold exposure for 3 days at 4°C. (B-D) qPCR analysis of thermogenesis-related genes in eWAT (B), iWAT (C), and BAT (D) from mice (n=5-6). Values were normalized to those of 36B4. Data are the mean ± SEM; *P<0.05, **P<0.01, ***P<0.001 between WT and KO mice.

Figure S3. (A) BrdU labelling with or without CL treatment. (B) Representative images of eWAT sections stained with adiponectin, PDGFRα, UCP1 and BrdU (red) in non-CL-treated mice. Arrows indicate BrdU^+^ cells expressing PDGFRα, adiponectin, and UCP1, respectively. Nuclei were counterstained with DAPI (blue). (C) The proportion of each cell type is expressed as a percentage of the total BrdU^+^ cells in non-CL-treated mice. *N.D*.; not detected. (D) Representative images of WAT sections stained with UCP1 (green) and BrdU (red) after CL treatment. Arrows mark UCP1^+^ BrdU^+^ cells. (E) Quantification of BrdU incorporation in UCP1^+^  cells in WAT after CL treatment. Data are the mean ± SEM, n=3; *P<0.05, **P<0.01, ***P<0.001 between WT and KO mice.

Figure S4. (A) qPCR analysis of thermogenesis-related genes in BAT from mice (n=5). Values were normalized to those of 36B4. (B) Immunoblot analysis of BAT from mice (n=5) treated with CL-316243. GAPDH was used as a loading control. (C) Immunoblot analysis of mitochondrial respiratory chain complexes in BAT of CL-treated mice (n=5). VDAC was used as a loading control. Data are the mean ± SEM; *P<0.05, **P<0.01, ***P<0.001 between WT and KO mice.

Figure S5. Relative mRNA levels of Adrb1 (A), Adrb2 (B), and Rgs2 (C) assessed by qPCR in adipose tissue of mice treated with CL316243 (n=5). Values were normalized to those of 36B4. (D) Levels of phospho-HSL and phosphorylated PKA substrates assessed by immunoblotting in BAT. (n=5). GAPDH was used as a loading control. Data are the mean ± SEM, *P<0.05, **P<0.01, ***P<0.001 between WT and KO mice.
